# Case Report: Atypical motor development in a patient with the mosaic form of Down syndrome and spinal muscular atrophy type 2- long-term observation

**DOI:** 10.3389/fgene.2024.1483903

**Published:** 2024-11-22

**Authors:** Ewa Gajewska, Jędrzej Fliciński, Magdalena Sobieska, Joanna Michalska, Marcin Żarowski, Barbara Steinborn

**Affiliations:** ^1^ Department of Developmental Neurology, Poznan University of Medical Sciences, Poznań, Poland; ^2^ Department of Rehabilitation and Physiotherapy, Poznan University of Medical Sciences, Poznań, Poland

**Keywords:** Down syndrome, spinal muscular atrophy, motor development, postural control, physiotherapy

## Abstract

A boy is presented in whom Down Syndrome mosaicism and spinal muscular atrophy by overlapping clinical symptoms delayed the diagnosis and caused complicated motor development. The boy from the first pregnancy was delivered vaginally, week 37, Apgar 10, birth weight 3,650 g. The mother, aged 30, had no family history of Down Syndrome or neuromuscular diseases. Primary diagnosis at the age of 3 weeks: unbalanced male karyotype -mos 47, XY+21 [22]/46, XY. At 20 months, the parents observed the disappearance of the high kneeling function and asked for a neurologist’s consultation. The neurological examination showed symmetrically reduced muscle tone and symmetrically weakened knee and ankle tendon reflexes. The physiotherapeutic assessment revealed a symmetrical weakening of muscle strength and hand tremor (features characteristic of patients with spinal muscular atrophy). The final diagnosis, set at the age of 27 months, was thus the mosaic form of Down Syndrome and spinal muscular atrophy type 2.

## 1 Introduction

Down Syndrome is the most common chromosomal abnormality in live-born individuals (about 1/800 live births), with 90%–95% of individuals with trisomy for chromosome 21. Mosaicism is seen in 2%–4% of individuals diagnosed with DS; affected individuals have both trisomic (47, XX+21 or 47, XY+21) and euploid (46, XX or 46, XY) cell lines (Papavassiliou et al., 2009).

A lower intensity of phenotypic dimorphic traits is characteristic of this form. In the case of mosaicism for trisomy 21, individuals have both trisomic and euploid cell lines. Mosaicism for trisomy 21 was first reported in 1961 by Clarke et al., who described an 11-month-old female who had good muscle tone, no congenital heart defects, and a normal development of milestones (Papavassiliou et al., 2015). Children with mosaicism attained developmental milestones earlier than non-mosaic individuals but later than chromosomally normal siblings (Papavassiliou et al., 2009). Children with mosaicism were described as following their own “idiosyncratic pattern of developmental progress (Fishler and Koch, 1991).

Spinal Muscular Atrophy (SMA) is the most common spinal motor neuron disease, occurring in 1 in 6–10,000 births with a carrier frequency of 1 in 35–70 (Ramsey et al., 2017). From 2005 to 2015, the incidence of SMA in Poland was estimated at 10.3–13.5/100,000 live births, while the average incidence in Europe from 2011 to 2015 was 11.9/100,000 (Bieniaszewska et al., 2022). SMA is an autosomal recessive condition, a deletion of the *SMN1* gene (Ramsey et al., 2017). The predominant clinical features of SMA are muscle weakness and atrophy, usually symmetric, with proximal muscles more affected than distal groups (Statland et al., 2015).

Patients experience gradual degeneration of alpha motoneurons, whose cell body is located in the cells of the anterior horns of the spinal cord, resulting in progressive muscle atrophy, areflexia in tendon reflexes, slowed motor development, contractures, or respiratory disorders (Gowda et al., 2023). The classification used previously for SMA patients was based on the child’s age at the onset of the first symptoms and the highest motor function achieved. Type 1 (SMA1; Werdnig- Hoffmann) comprises patients who never reach the function of sitting up on their own, with the early (first months of life) onset of symptoms. Type 2 (SMA2) refers to the sedentary patients diagnosed between 6 and 18 months of age who will never achieve independent walking. The mild type (SMA3; Kugelberg-Welander) comprises children whose highest function is independent walking, and the onset of symptoms occurs after 18 months of age (Stępień et al., 2020; Verhaart et al., 2017; Chong et al., 2021). As new therapies were introduced for SMA patients, children described as type I, unable to walk nor sit, may now achieve higher skills such as standing and walking (Wang et al., 2007; Mercuri et al., 2018; Finkel et al., 2018). Therefore, a new classification describes children as non-sitter, sitter, or walker, which are defined by the patient’s functional level (Trenkle et al., 2021). Spinal muscular atrophy manifests as muscle flaccidity and gradual atrophy, resulting in progressing functional limitations. Motor development is known to regress in untreated patients. Characteristics and symptoms of SMA include the loss of previously acquired motor function in children, with the addition of respiratory distress, dysphagia, and joint contractures (Bieniaszewska et al., 2022).

The presented boy is affected by two independent genetic disorders, causing the mosaic form of Down Syndrome (DS) and spinal muscular atrophy (SMA). We present a situation in which the coexistence of two diseases by overlapping clinical symptoms may affect the delay of a diagnosis. Due to such a complicated cause, his motor development is atypical and seems worth presenting from the point of view of a physiotherapist.

## 2 Case presentation

A 23-month-old boy was referred by a neurologist for rehabilitation due to reduced muscle tone and delayed and receding motor development.

The boy from the first pregnancy was delivered vaginally, week 37, Apgar 10, birth weight 3,650 g. The mother, aged 30, had no family history of DS or neuromuscular diseases. During the pregnancy, the double test was performed according to the method of the Fetal Medicine Foundation, and the test result was negative.

Due to phenotypic traits at 3 weeks, the boy was subjected to a genetic test which showed abnormal, unbalanced male karyotype - mosaic 47, XY+21 [22]/46, XY.

Motor development in the first year of life was uneven. He could roll from prone to supine at 6 months (normal for age) and finally assumed a crawling position at 8 months. Crawling began in the 10th month of life. In the same month, the boy started to reach the sitting position and sit independently. In the 11th month of life, the boy could assume the high kneeling position next to furniture.

At the age of 20 months, parents observed a disturbing symptom: the disappearance of the high kneeling function and asked for a neurologist’s consultation. The neurological examination showed symmetrically reduced muscle tone and symmetrically weakened knee and ankle tendon reflexes. The neurologist referred the patient for rehabilitation.

Patients with mosaic trisomy may experience delayed psychomotor development compared to their peers, but there is no regression in motor development, which occurs and is characteristic of spinal muscular atrophy.

It is particularly important to mention that regression/loss of function does not occur in the course of motor development in children suffering from Down Syndrome, even in mosaic forms. Therefore, if such a feature is observed, a child should be assessed in detail.

The child therapist who assessed the child at 21 months had experience in functional assessment of children with SMA and observed that the boy, in addition to the irregularities reported by the parents and neurologist, also showed a symmetrical weakening of muscle strength and hand tremors (features characteristic of patients with SMA). Based on this observation, at the age of 27 months, the neurologist referred the boy for additional genetic testing for SMA. At the same time, physiotherapy was initiated to improve/maintain the functions achieved. The *SMN1* and *SMN2* genes were analyzed in the genetic test, and the presence of homozygous deletion of exons 7 and 8 of the *SMN1* gene (absence of both copies of exons 7 and 8) was found. In addition, heterozygous duplication of exons 7 and 8 of *SMN2* was detected (three copies of exons 7 and 8 of the *SMN2* gene). The final diagnosis was thus the mosaic form of DS and SMA2.

The patient’s brother, who is 19 months younger, underwent a neurological examination at the age of 8 months. The examination found a symmetrical reduction of muscle tone and the absence of knee reflexes. The patient’s brother was referred for genetic testing, which also showed a diagnosis of SMA2.

Functional evaluation of the patient was complex because, due to the overlap of two genetic abnormalities, his motor development was not typical of the initially diagnosed mosaic DS, pointing more to deficits associated with SMA. Only after the second diagnosis (at 27 months) were specific tools for SMA assessment used, such as a modified Hammersmith scale. We know this tool was not validated for such an unusual form, but because typical features of SMA prevail in the patient in question, this was an assessment method of choice (Krosschell et al., 2006). The authors of the scale made reliability and sensitivity data for all children younger than 30 months assessed and compared to reliability and sensitivity data for all children above 30 months of age. It was determined that age was not a factor. However, in the youngest children, delayed achievement of motor milestones could theoretically lead to improvements in scores that are not treatment-related but a function of developmental maturation (Krosschell et al., 2006). A review of the developmental literature suggests that correctly developing children achieve all items on the scale before 20 months (Krosschell et al., 2006).

The patient’s motor development was analyzed retrospectively (based on the medical documentation provided) at the age of 12 months (first concern) and 20 months (when motor development continued to be at a standstill and the patient lost the previously achieved high kneeling function), and further prospectively: at the age of 28, 30 months; 4, 6, 7 years (Table 1). According to the new classification, the patient should be described as a sitter (Trenkle et al., 2021).

**TABLE 1:** Analysis of functional changes using the modified Hammersmith scale.

Modified Hammersmith scale, pre-treatment assessment					Modified Hammersmith scale, post-treatment evaluation		
Function under assessment	12 months	20 months	28 months	30 months	4 years	6 years	7 years
Frog (floor)/chair sitting no hand support	2	2	2	2	2	2	2
Long sitting, no hands	2	2	2	2	2	2	2
Raises one hand to ear level in sitting R/L	2/2	2/2	2/2	2/2	2/2	2/2	2/2
Raises 2 hands to ear level (in sitting)	2	2	2	2	2	2	2
Able to assume the lying position from sitting (safely, not accidently)	2	2	2	2	2	2	2
Lifts head from the surface in supine position	2	2	0	0	0	2	2
1/2; Rolls from the supine position, both ways	2	2	2	2	2	2	2
Rolls from the prone to supine position over R	2	2	2	2	2	2	2
Rolls from the prone to supine position over L	2	2	2	2	2	2	2
Rolls from the supine to prone position over R	2	2	2	2	2	2	2
Rolls from the supine to prone position over L	2	2	2	2	2	2	2
Lifts head from the prone position (arms down by sides)	2	2	2	2	2	2	2
Achieves prop on forearms-head up	2	2	2	2	2	2	2
Achieves prop on extended arms-head up	2	2	2	1	2	2	2
Achieves four point kneeling	2	2	2	1	2	2	2
Crawls on hands and knees	2	2	0	0	1	2	2
Assumes the sitting position from lying through side lying	2	2	1	1	1	2	2
Stands holding on with one hand	0	0	0	0	0	0	0
Stands independently: count >3	0	0	0	0	0	0	0
Takes >4 steps independently	0	0	0	0	0	0	0

At 12 and 20 months, the following functions were reported to be missing: The ability to stand holding on with one hand, to stand independently: count >3, and to take >4 steps independently.

In addition, constipation was reported and treated pharmacologically, while respiratory disorders were not found.

At 28 months, the patient had lost the crawling function and could not lift his head in the supine position, assuming the sitting position from lying through side lying-rated one point. Daily rehabilitation focused on maintaining motor activities. At 30 months, due to the high dynamics of the loss of function, the patient, compared to the last test, performed poorly in the following way: achieving a point kneeling position and prop on extended arms-head up. Still, but less frequently, the patient manifested the ability to sit from the lying position though lying on the side, performed slowly and with visible difficulty (Table 1). He preferred to be seated in the sitting position by the parent. He preferred to rotate rather than creep as a method of moving towards toys. In addition, the ranges of movement in the joints were checked. No contractures, which gradually occur in patients with SMA, were reported.

A definite improvement in the development of social interactions, e.g., speech, understanding speech (the patient performed complex commands), or games, was shown, and a slight delay was reported compared to the development standards. No disorders associated with the respiratory function were found. In May 2019 (at the age of 34 months), Nusinersen treatment was started. Table 1 shows the patient’s development after the treatment at 4, 6, and 7 years.

## 3 Discussion

There are no publications on the motor development of children with the mosaic form of Down Syndrome. It can only be guessed that depending on the cell development line affected by trisomy, a different effect on the functioning, including motor development, may be observed, and abnormalities typical of the DS will usually be less pronounced than in the full-blown syndrome. In children with DS, these motor deficits reflect specific characteristics: balance, posture, strength, and motor planning, which are found to be particularly weak and are interwoven with their intellectual functioning (Marchal et al., 2016).

In the case of the patient in question, one should also consider that intellectual deficits typical of DS affect motor development. Still, the loss of function should be attributed to a much greater extent to muscular disorders typical of SMA2.

In 1991, three types of SMA were distinguished, depending on the highest functional level (i.e., sitting or standing) and the age of onset (Zerres and Davies, 1999; Dubowitz, 2019). Later, type 4 for adults and 0 for the rare form were added, where the symptoms were visible as early as in the fetal period (Glanzman et al., 2010; Kolb and Kissel, 2015). Despite individual differences, this scheme remains relevant and provides useful clinical and prognostic information (Kaufmann et al., 2011). As a result of the introduction of treatment, this classification has been replaced by new phenotypes, such as non-sitters, sitters, and walkers, which are defined by the patient’s functional level.

This work focuses on SMA type 2 (sitter) because this form was diagnosed in the patient under study. SMA type 2 accounts for approximately 20% of cases, manifests between 3 and 15 months of age, and patients are diagnosed between 6 and 18 months of age; the highest level of motor skills achieved is independent sitting (Zerres and Rudnik-Schöneborn, 1995; Finkel et al., 2015). In the patient in question, the test for SMA was performed much later, at the age of 27 months.

We retrospectively observed the patient’s motor development at 12 and 20 months; the subsequent analysis was performed prospectively. Due to the patient’s age and the possibility of using the Hammersmith Functional Motor Scale (O’Hagen et al., 2007), we used the modified Hammersmith functional motor scale (Krosschell et al., 2006).

Rudnik-Schoneborn et al. showed that 73% out of 175 patients with SMA type 2 maintained the sitting position for up to 9 months, while the rest achieved this function between 10 and 30 months of life (Rudnik-Schöneborn et al., 2001). This function was reached early, at 10 months, in the patient in question.

Kaufman et al. observed that in 35 out of 71 patients with SMA type 2 or 3 (no treatment), no loss of motor function measured with GFM, HMFS, or ExpHFMA was detected during the 12-month follow-up, suggesting a stable, non-progressive course of the disease (Kaufmann et al., 2012). Studies also show that the motor function of patients with SMA type 2 or 3 can improve (Dunaway et al., 2012).

Regrettably, a very significant loss of motor function during the 18-month follow-up was shown in the patient in question. After 16 months, the patient failed to crawl on his hands and knees and lift his head from the surface in the supine position. In contrast, after two consecutive months, he performed poorly on the following functions: achieving prop on extended arms with head up, achieving the four-point kneeling, and assuming the sitting position from lying through side lying.

After starting Nusinersen treatment, the patient improved his functions, lifting his head from the surface in the supine position, achievement of prop on extended arms-head up, achievement of the four-point kneeling, crawls on hands and knees and better-doing achievement of prop on extended arms-head up, achievement of the four-point kneeling and assuming the sitting position from lying through side lying. The drug modulates alternative splicing of the SMN2 gene, functionally transforming it into the SMN1 gene, thereby increasing SMN protein levels in the central nervous system (Pao et al., 2014).

Motor assessment of children with Down Syndrome can be based on the use of the Gross Motor Function Measure or Alberta Infant Motor Scale; for children with SMA, the CHOP-Intend, Hammersmith Functional Motor Scale, or Motor Function Measure scales are typically used, depending on the highest function achieved. There are no guidelines for children with an overlap between these two mutations, but due to the predominant phenomenon of regression of function, the patient was evaluated like children with SMA, considering his functional level as a sitter.

The late diagnosis of SMA type 2 was influenced by the earlier diagnosis of the mosaic form of DS and the fact that the course of the disease was not typical, neither for DS nor SMA. The concern of the neurologist and the physiotherapist was raised by regression in motor development, loss of knee reflexes, muscle strength weakening, more severe symptoms in the lower extremities than in the upper extremities, tremors, and lack of progress in the development despite intense physiotherapy. The reduced muscular tone was initially attributed only to DS. Unfortunately, there are no reports on this issue regarding the mosaic form of DS. However, the therapist’s experience regarding work with children suffering from SMA points to the fact that incorrect postural control, and especially the loss of the previously achieved functions, raises the suspicion of another genetic basis of the observed disorders, which had been confirmed by genetic research.

## 4 Conclusion

To date, there is no account in the literature of the assessment of motor function development of a patient with the mosaic form of DS and SMA. An additional difficulty is that defects associated with both genetic abnormalities affect the same elements of the motor system, and it is impossible to determine which disorders are associated with which syndrome. Based on the experience obtained from this case, we can say that any change in the expected development merits a new multidisciplinary approach.

## Data Availability

The raw data supporting the conclusions of this article will be made available by the authors, without undue reservation.
